# Serum Tryptase Monitoring in Indolent Systemic Mastocytosis: Association with Disease Features and Patient Outcome

**DOI:** 10.1371/journal.pone.0076116

**Published:** 2013-10-14

**Authors:** Almudena Matito, José Mario Morgado, Iván Álvarez-Twose, Laura Sánchez-Muñoz, Carlos Eduardo Pedreira, María Jara-Acevedo, Cristina Teodosio, Paula Sánchez-López, Elisa Fernández-Núñez, Ricardo Moreno-Borque, Andrés García-Montero, Alberto Orfao, Luis Escribano

**Affiliations:** 1 Instituto de Estudios de Mastocitosis de Castilla-La Mancha, Hospital Virgen del Valle, Toledo, Spain; 2 COPPE-Instituto Alberto Luiz Coimbra de Pós-Graduaçäo e Pesquisa de Engenharia and Federal University of Rio de Janeiro, Rio de Janeiro, Brazil; 3 Servicio General de Citometría, Centro de Investigación del Cáncer (IBMCC-CSIC/USAL) and Departamento de Medicina, Universidad de Salamanca, Salamanca, Spain; 4 Allergy Department, Hospital Rey Juan Carlos, Móstoles, Spain; 5 Spanish Network on Mastocytosis (REMA); Institut national de la santé et de la recherche médicale (INSERM), France

## Abstract

**Background:**

Serum baseline tryptase (sBT) is a minor diagnostic criterion for systemic mastocytosis (SM) of undetermined prognostic impact. We monitored sBT levels in indolent SM (ISM) patients and investigated its utility for predicting disease behaviour and outcome.

**Methods:**

In total 74 adult ISM patients who were followed for ≥48 months and received no cytoreductive therapy were retrospectively studied. Patients were classified according to the pattern of evolution of sBT observed.

**Results:**

Overall 16/74 (22%) cases had decreasing sBT levels, 48 (65%) patients showed increasing sBT levels and 10 (13%) patients showed a fluctuating pattern. Patients with significantly increasing sBT (sBT slope ≥0.15) after 48 months of follow-up showed a slightly greater rate of development of diffuse bone sclerosis (13% vs. 2%) and hepatomegaly plus splenomegaly (16% vs. 5%), as well as a significantly greater frequency of multilineage vs. mast cells (MC)-restricted *KIT* mutation (p = 0.01) together with a greater frequency of cases with progression of ISM to smouldering and aggressive SM (p = 0.03), and a shorter progression-free survival (p = 0.03).

**Conclusions:**

Monitoring of sBT in ISM patients is closely associated with poor prognosis disease features as well as with disease progression, pointing out the need for a closer follow-up in ISM patients with progressively increasing sBT values.

## Introduction

Systemic mastocytosis (SM) includes a heterogeneous group of disorders with increased mast cell (MC) numbers in different organs and tissues [1], [2]. The clonal nature of mastocytosis can be established in virtually every case through demonstration of exon-17 *KIT* mutations in MC [3], [4], except for a rare subset of well-differentiated SM patients [3]–[8]. From the clinical point of view, the presence of multilineal *KIT* mutation in multiple bone marrow (BM) hematopoietic lineages (e.g. myeloid and lymphoid BM cells) represents in the largest series of ISM reported so far, the most relevant prognostic factor of the disease, since it is able to identify those patients at risk of progression to more advanced forms of the disease [9].

Inside their cytoplasmic granules, MC contain tryptases (EC 3.4.21.59), a group of up to four distinct proteases (α-tryptase, β-tryptase, δ-tryptase and γ-tryptase) which are also present in smaller quantities in blood basophils [10]. Although all four forms of tryptase are coded by genes localized in chromosome 16 [11], [12]; they show a different pattern of expression in serum. Thus, δ-tryptase is not detected in serum and γ-tryptase is not released in a soluble form [13]. In turn, β-tryptase is stored in the secretory granules of MC as an enzymatically active tetramer forming a complex with heparin, which is released after MC activation, whereas in vitro generation of α-(pro)tryptase is associated with the formation of enzymatically inactive tetramers [14], [15], and it is constitutively released from MC into the plasma [16].

Currently, the only commercial technique available for the quantification of total tryptase is ImmunoCAP Tryptase System (Phadia, Uppsala, Sweden/Thermo Fisher Scientific Inc.); using this test system, total tryptase values can be determined, although the different isoforms of tryptases cannot be differentiated. Increased total serum baseline tryptase (sBT) -recorded in the absence of acute MC mediator release episodes- has long been described in ISM [16], [17], and sBT levels >20 ng/mL is a (minor) diagnostic criterion for SM [1], [2]. Overall, sBT levels in SM have been associated with the total body MC burden [18], the extent of BM involvement [1], diffuse bone sclerosis [8], [9], [19], and the diagnostic subtype of the disease [20]–[22]. However, markedly increased sBT have also been found in disease conditions other than mastocytosis such as anaphylactic episodes [16], [23] associated or not with hymenoptera venom sting [24], clonal myeloid malignancies [22], a subset of hypereosinophilic syndromes [25], chronic urticaria [26] and advanced kidney disease [27]–[29]. Despite all the above, no study has been reported so far in which the utility of sBT monitoring has been investigated in ISM patients to determine its potential utility to predict for the behaviour of the disease and patient outcome.

Here, we investigated the association between the pattern of evolution of sBT levels during the first 48 months of follow-up in ISM and adverse disease features, prognostic factors, as well as progression of ISM to more advanced subtypes of the disease (e.g. smouldering -SSM- and aggressive SM -ASM-).

## Materials and Methods

This work was included in the research project RETICS RD09/0076/00074, Hospital Virgen de la Salud Biobank (BioB-HVS), Toledo, Spain; so, the approval was obtained from the institutional review board “Comité Ético de Investigación Clínica del Complejo Hospitalario de Toledo”. It was completely conducted in Spain, and all the adult participants as well as the caretakers of the minors participants provided their written informed consent to participate.

### Patients

A total of 74 adults diagnosed with ISM who have been followed by the Spanish Network on Mastocytosis (REMA) between March 1995 and September 2012, were included in this study. Inclusion criteria were: i) follow-up ≥48 months; ii) ≥3 sBT determinations recorded in basal clinical situations during this period; iii) absence of diffuse bone sclerosis at first sBT determination;^(19)^ iv) absence of cytoreductive or targeted therapies that may had induced a decrease in sBT, and; v) data available on *KIT* mutational status for highly-purified bone marrow mast cells (BMMC), as well as purified cells from other hematopoietic cell lineages. From the 74 patients analyzed, 33 (45%) were men and 41 (55%) women - median age at both the first sBT determination and the first BM study of 41 years (range: 15–72 and 16–74 years, respectively)-. Diagnosis of ISM was performed following previously established criteria for morphology [30], histopathology and immunohistochemistry [31], flow cytometry immunophenotyping [6], [8], [32]–[35], and molecular detection of *KIT* mutations [3] following the World Health Organization (WHO) criteria [1].

A clinical and physical work-up together with a routine peripheral blood (PB) count and differential, routine biochemistry and sBT (ImmunoCAP Tryptase System, Phadia, Uppsala, Sweden/Thermo Fisher Scientific Inc.) were performed at referral and thereafter, every 6 to 15 months. At the moment of closing this study, all cases remained alive after a median follow-up of 113 months (range: 51–193 months), and a total of 787 sBT determinations -median of 9 (range: 3–24) measurements per patient- were performed. During follow-up, imaging studies including abdominal ultrasonography and/or computed tomography scan (CTscan), dual energy X-ray absorptiometry, skeletal X-ray survey and/or magnetic resonance imaging (MRI) were performed every 2 years, except in cases suspicious of having clinical progression. Osteoporosis was defined following well-established criteria [36] and the presence of bone sclerosis was assessed by x-ray of bones, CTscan, and/or MRI. The presence of B and C findings, as well as smouldering SM (SSM) as defined by the WHO criteria [1] were recorded in each case.

Treatment was selected based on the intensity and/or severity of symptoms. Different drugs (alone or in distinct combinations) were used following previously described criteria [37]. Therapy consisted of: i) the MC-stabilizer oral disodium cromolyn since the referral; ii) scheduled or at demand sedating H1-antihistamines (dexchlorpheniramine); iii) scheduled or at demand non-sedating H1-antihistamines, depending on their availability along the study; iv) scheduled H2-antihistamines, and; v) scheduled leukotriene antagonists; vi) corticosteroids and epinephrine were only administered when strictly required. Other therapies used in selected patients included non-steroidal anti-inflammatory agents for refractory abdominal cramping and diarrhoea and, in non-responders, short cycles of either low doses of prednisone (0.3 mg/Kg/day) or oral budesonide (0.1 mg/Kg/day), with progressively decreasing doses. In stress-induced anaphylaxis, a psychiatric work-up was performed and adequate anxiolytic and/or anti-depression therapy used. Intensive antimediator therapy was defined by the use of scheduled disodium cromolyn plus any of the other referred drugs to control MC-mediator related symptoms. Cytoreductive therapy (interferon alpha-2b, hidroxyurea and cladribine) was prescribed in only 2 cases, following progression to aggressive SM (ASM); further sBT determinations performed in these two cases were excluded from the study. Disease progression was defined as transformation of ISM into a more aggressive WHO subtype of mastocytosis (e.g.SSM and ASM) [1], [2].

### Statistical methods

K-means clustering analysis was used to classify ISM patients into three groups according to the pattern of evolution of their sBT levels during the first 48 months of follow-up: i) decreasing; ii) fluctuating (increasing followed by decreasing sBT levels and vice versa), and; iii) continuously increasing sBT. In addition, variation in sBT was estimated by using a linear regression model ("y = ax+b") to establish the relationship between sBT levels ("y") and the time of follow-up of the disease ("x"), and to calculate time-associated changes in sBT levels ("a" being calculated as the slope of the best linear fit), reflecting stable or decreasing versus increasing sBT levels: slope of the best linear fit ≤0 (e.g. negative) or >0 (e.g. positive), respectively.

The Kruskall Wallis and the Mann-Whitney U, or the χ^2^ tests were used to assess the statistical significance of differences observed between groups for continuous and categorical variables, respectively. For multivariate analyses (logistic regression), only those variables that showed a statistically significant association in the univariate study, were included in the model.

Optimal cut-off values for the slope of the best linear fit of sequential sBT levels for predicting for multilineal *KIT* mutation were calculated by receiver operating characteristic (ROC) curves. Progression-free survival curves were plotted according to the method of Kaplan and Meier and compared by the log-rank test. Statistical significance was set at p values <0.05. For all statistical analyses, the SPSS 17.0 (SPSS, Chicago, Ill), Excel 2010 (Microsoft) and MatLab R2010a (The MathWorks, Inc.) software packages, were used.

## Results

### Pattern of evolution of disease features

According to the pattern of evolution of sBT levels during the first 48 months of follow-up, 16 (22%) cases had decreasing sBT levels, 10 (13%) patients showed a fluctuating pattern of sBT with increasing (n = 5) or decreasing (n = 5) levels in the last analysis performed, and the remaining 48 (65%) cases showed increasing sBT levels, with either a continuous increase –20 (27%) cases- or an initial increase followed by a tendency to stabilization –28 (38%) patients-. No significant differences were observed among these three groups of patients as regards disease features at diagnosis, except for a greater frequency of multilineal *KIT* mutation (p = 0.02) and disease progression among cases with increasing sBT levels (Table 1).

**Table 1:** Demographic, clinical and laboratory characteristics of ISM patients (n = 74) grouped according to the pattern of evolution of sBT after 48 months of follow-up.

	sBT pattern			
Variable	DECREASING (n = 16)	FLUCTUATING (n = 10)	INCREASING (n = 48)	p value
Female patients*	11 (69%)	5 (50%)	25 (52%)	NS
Age at onset of the disease (years)	29 (7 – 52)	25 (0 – 43)	29 (12 – 72)	NS
Age at 1^st^ BM study (years)	43 (16 – 62)	33 (17 – 48)	42 (22 – 74)	NS
Age at 1^st^ sBT determination (years)	43 (16 – 62)	32 (17 – 48)	41 (15 – 72)	NS
Time from disease onset to 1^st^ BM study (months)	135 (27 – 463)	101 (45 – 210)	71 (0 – 281)	NS
Time from disease onset to first sBT determination (months)	104 (27 – 463)	99 (45 – 210)	62 (0 – 88)	NS
Time from 1^st^ sBT determination to 1^st^ BM study (months)	2 (0 – 71)	2 (0 – 12)	0 (0 – 91)	NS
Time of follow-up (months)	118 (61 – 170)	90 (56 – 146)	117 (51 – 193)	NS
First sBT determination (ng/mL)	39.7 (8.7 – 199)	22 (12.5 – 114)	26.6 (2.5 – 148)	NS
Last sBT determination (ng/mL)	19.2 (7 – 183)	25.6 (10.7 – 70.8)	44 (7 – 1300)	NS
sBT slope after 48 months of follow-up	-0.23 (-2 – 0.04)	0.01 (-0.44 – 0.14)	0.25 (-0.09 – 12.4)	<0.001†
sBT slope ≥0.15 after 48 months of follow-up *	0 (0%)	0 (0%)	31 (65%)	<0.001
Multilineal *KIT* mutation*	0 (0%)	2 (20%)	17 (35%)	0.02
SSM*	0 (0%)	0 (0%)	4 (8%)	NS
Progression to ASM *	0 (0%)	0 (0%)	2 (4%)	NS

Results expressed as median values and range between brackets and *as number (percentage) of cases.

ASM, aggressive systemic mastocytosis; BM, bone marrow; NS, not statistically significant (p >0.05); sBT, serum baseline tryptase; SSM, smouldering systemic mastocytosis.

† DP vs. FP, p = 0.008; DP vs. IP, p<0.001; FP vs. IP, p<0.001.

All cases with decreasing as well as fluctuating tryptase levels, showed a sBT slope of <0.15; in more detail, cases with decreasing sBT constantly displayed a negative slope while the group with a fluctuating pattern included cases with both positive and negative sBT slopes (5 and 3 cases, respectively). Conversely, most (31/48) cases with increasing tryptase levels had a slope ≥0.15; among the other 17 cases with increasing tryptase levels, 12 patients showed a positive slope but with values lower than 0.15. (Table 1 and Table 2).

**Table 2:** Demographic, clinical and laboratory characteristics of ISM patients grouped according to the pattern of changes in sBT levels (sBT slope) after 48 months of follow-up.

Variable	sBT slope < 0.15 (n = 43)	sBT slope ≥ 0.15 (n = 31)	p value
Female patients	27 (63%)	14 (45%)	NS
Age at onset of the disease (years) *	28 (0 – 61)	29 (12 – 72)	NS
Age at 1^s^t BM study (years) *	41 (16 – 63)	42 (22 – 74)	NS
Age at 1^st^ sBT determination (years) *	41 (16 – 63)	42 (15 – 72)	NS
Time from disease onset to 1^st^ BM study (months) *	84 (0 – 463)	71 (0 – 254)	NS
Time from disease onset to 1^st^ sBT determination (months) *	84 (0 – 463)	64 (0 – 254)	NS
Time from 1^st^ sBT determination to first BM study (months) *	1 (0 – 71)	0 (0 – 91)	NS
Time of follow-up (months) *	118 (59 – 193)	105 (51 – 191)	NS
Skin lesions	39 (91%)	28 (90%)	NS
Multilineal *KIT* mutation	6 (14%)	13 (42%)	0.01
Percentage of BMMC*†	0.07 (0.004 – 0.7)	0.1 (0.001 – 1.7)	NS
Osteoporosis at first evaluation	4 (9%)	3 (10%)	NS
Osteoporosis at the end of follow-up	6 (14%)	5 (16%)	NS
Patchy bone sclerosis at the end of follow-up	2 (5%)	2 (6%)	NS
Diffuse bone sclerosis at the end of follow-up	1 (2%)	4 (13%)	NS
Organomegaly at first evaluation	0 (0%)	1 (3%)‡	NS
Organomegaly at the end of the follow-up	3 (7%)§	6 (19%)∣∣	NS
Progression to SSM	0 (0%)	4 (13%)	0.03
Progression to ASM	0 (0%)	2 (6%)	NS

Results expressed as number of cases from total cases in the group and percentage between brackets or *as median values and range between brackets.

ASM, aggressive systemic mastocytosis; BM, bone marrow; ISM, indolent systemic mastocytosis; NS, not statistically significant; MC, mast cells; sBT, serum baseline tryptase; SSM, smouldering systemic mastocytosis.

† Assessed by flow cytometry.

‡ Splenomegaly.

§ Hepatomegaly in one case, hepatomegaly plus splenomegaly in the other 2 patients.

∣∣Splenomegaly in one case, hepatomegaly plus splenomegaly in the other 5 patients.

Upon regrouping ISM patients into cases presenting with significantly increasing sBT levels ≥15% after 48 months of follow-up (slope ≥0.15) vs. all other cases (sBT slope <0.15) there were no significant differences between the two patients groups as regards their demographic and follow-up characteristics (Table 2) except again, for a greater frequency of multilineal *KIT* mutation (42% vs. 14%, p = 0.01%) and disease progression (13% vs. 0%, p = 0.03) among cases with a higher sBT slope.

The frequency of MC-mediator related symptoms and the usage of antimediator therapies were also similar in both groups (Table 3). In addition, cases with a sBT slope ≥0.15 after 48 months of follow-up showed similar frequencies to those found among the other patients (p>0.05) of osteoporosis as well as of patchy and diffuse bone sclerosis, both at presentation and at the end of follow-up (Table 2). Despite this, diffuse bone sclerosis slightly developed more frequently among cases having a sBT slope ≥0.15 (13% vs. 2% respectively, p>0.05). Similarly, organomegalies (hepatomegaly plus splenomegaly) also developed slightly more frequently among ISM cases with a sBT slope ≥0.15 (16% vs. 5%, p>0.05).

**Table 3:** Therapies used to control symptoms related to the release of mast-cell mediators in ISM patients grouped according to the sBT slope (<0.15 vs. ≥0.15) after 48 months of follow-up.

	sBT slope <0.15 (n = 43)			sBT slope ≥0.15 (n = 31)		
Antimediator therapy	At referral	48 months of follow-up	End of follow-up	At referral	48 months of follow-up	End of follow-up
Scheduled disodium cromolyn	7 (16%)	34 (79%)	38 (88%)	5 (16%)	27 (87%)	27 (87%)
Scheduled intensive antimediator therapy*	0 (0%)	12 (28%)	11 (26%)	0 (0%)	12 (39%)	12 (39%)
Antimediator therapy at demand†	13 (30%)	9 (21%)	6 (14%)	8 (26%)	5 (16%)	6 (19%)
Epinephrine	3 (7%)	2 (5%)	1 (2%)	3 (10%)	1 (3%)	0 (0%)

Results are expressed as number of cases and percentage between brackets.

No statistically significant differences (p >0.05) were found between groups.

* Scheduled disodium cromolyn plus scheduled non-sedating or sedating H1 and/or H2 antihistamines, and/or leukotriene antagonists, and/or anxiolytic and/or anti-depression therapy.

† Non-sedating or sedating H1 antihistamines and/or corticosteroids and/or epinephrine.

### sBT monitoring and disease outcome

Overall, 4 cases developed SSM after a median follow-up of 49 months (range: 8–85 months). All four patients reached sBT values >200 ng/mL after a median time of 57 months (range: 35–85 months), and they all had both sBT slopes ≥0.15 (p = 0.03; Table 2) and multilineal *KIT* mutation (p = 0.003). In addition, two of them progressed to aggressive SM (ASM) after 56 and 94 months of follow-up; both had an increasing sBT pattern with a sBT slope of 12.44 and 0.98, respectively; furthermore, both cases developed diffuse bone sclerosis (after 37 and 90 months) together with hepatomegaly plus splenomegaly (after 54 and 90 months).

**Figure 1:**
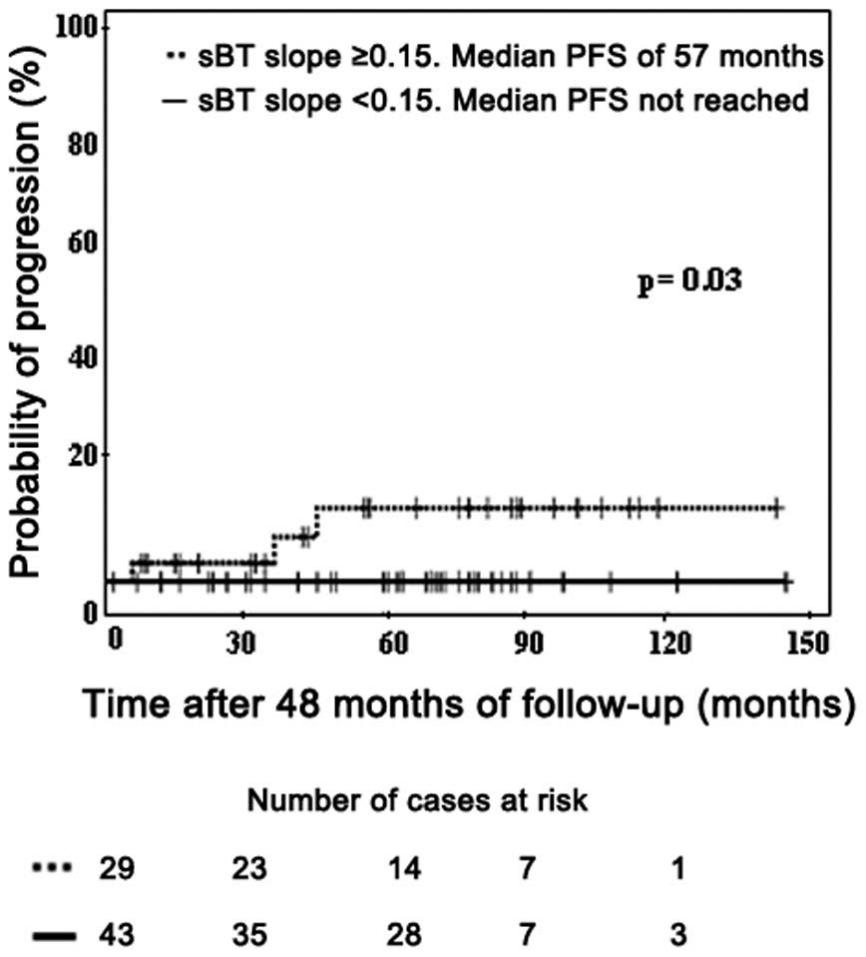
Impact of the pattern of evolution of sBT levels (sBT slope) in progression-free survival of ISM patients. PFS, progression-free survival; sBT, serum basal tryptase.

From a prognostic point of view, increasing sBT (slope ≥0.15) together with the development of hepatomegaly plus splenomegaly, were the only two informative parameters to predict for multilineal involvement of BM cells by the *KIT* mutation (p = 0.01, and p = 0.009, respectively; Table 4); furthermore, multivariate analysis confirmed that both parameters were independent and that they provide the best combination of factors to predict for multilineal *KIT* mutation with a 100% specificity (19% false-negative and 0% false-positive results; Table 4). Similarly, increasing sBT (slope ≥0.15) was a relevant predictor for progression of ISM to more advanced forms of the disease such as SSM and ASM, (p = 0.03; Figure 1).

**Table 4:** Univariate and multivariate analysis of predictive factors for the presence of multilineal *KIT* mutation.

	Univariate analysis			Multivariate analysis	
Predictive factor	Patients, No. (%)	RR (95% CI)	p value	HR (95% CI)	p value
sBT slope ≥0.15 after 48 months of follow-up	13 (42%)	4.5 (1.5 – 13.6)	0.009	3.8 (1.2 – 12.3)	0.02
Hepatomegaly plus splenomegaly	5 (71%)	9.5 (1.7 – 54)	0.01	7.4 (1.2 – 45.8)	0.03

CI, confidence interval; HR, hazard ratio; RR, relative risk.

## Discussion

sBT is a useful biomarker for the diagnosis of SM including ISM. However, few studies have analyzed the potential prognostic value of sBT monitoring for the identification of ISM cases at risk of disease progression. In the present study we investigated the potential association between the pattern of evolution of sBT levels during the first 48 months of follow-up and the features of the disease both at diagnosis and during follow-up, including progression to more aggressive forms of systemic mastocytosis. Overall our results show that an increase in sBT levels after 48 months of follow-up with a slope ≥0.15 is associated with both the presence of multilineal *KIT* mutation, and progression of ISM to SSM (and to a less extent also ASM). Based on the cut-off value of 0.15 for the slope of sBT levels during the first 48 months of follow-up, a major group of patients who systematically show *KIT* mutation restricted to MC and stable disease together with a smaller subset of ISM patients who more frequently show multilineal *KIT* mutation (a parameter that has been associated with a greater risk of disease progression) [9], [38] were identified. In addition to sBT, the presence of hepatomegaly plus splenomegaly also emerged as an independent predictor for multilineal *KIT* mutation, and only this subset of multilineal cases developed diffuse bone sclerosis; these results suggest the existence of a close association between these two features. In addition, sBT levels also showed a significant impact on progression-free survival of ISM patients, despite the still relatively limited follow-up. Of note, sBT levels were systematically assessed in basal clinical conditions, to avoid transiently elevated sBT levels associated to acute MC-mediators release episodes, and no clear relationship was observed between the use of antimediator therapy and the overall behaviour of sBT (evaluated by the slope of sBT levels measured during the first 48 months of follow-up). Despite this, we can not fully rule out an impact of therapy (e.g. admisnistration of disodium cromolyn) on decreasing MC-mediators release in individual patients, particularly in those cases showing a negative sBT slope; further investigations are required in this regard.

Previous reports have shown that multilineage involvement of myeloid and/or lymphoid cells by the D816V *KIT* mutation is the most powerful predictor for disease progression in ISM [9]; however, specific investigation of *KIT* mutation in multiple individual myeloid and lymphoid compartments of maturing BM hematopoietic cell lineages, is a technically demanding approach which requires bone marrow sampling and is currently not routinely performed in most diagnostic laboratories. In addition, an immature immunophenotype of BMMC (defined by the aberrant expression of CD25, usually in the absence of CD2, associated with decreased expression of CD117, FcεRI and increased positivity for CD123, HLA-DQ, and HLA-DR) could also predict for multilineal *KIT* mutation in ISM, regardless of the diagnostic subtype of the disease [38]; however, such approach is also frequently not available in many centres. Therefore, from a practical point of view monitoring of sBT emerges as a potentially interesting candidate surrogate marker for multilineage *KIT* mutation among ISM with higher risk for progression to more advanced forms of the disease.

**Figure 2:**
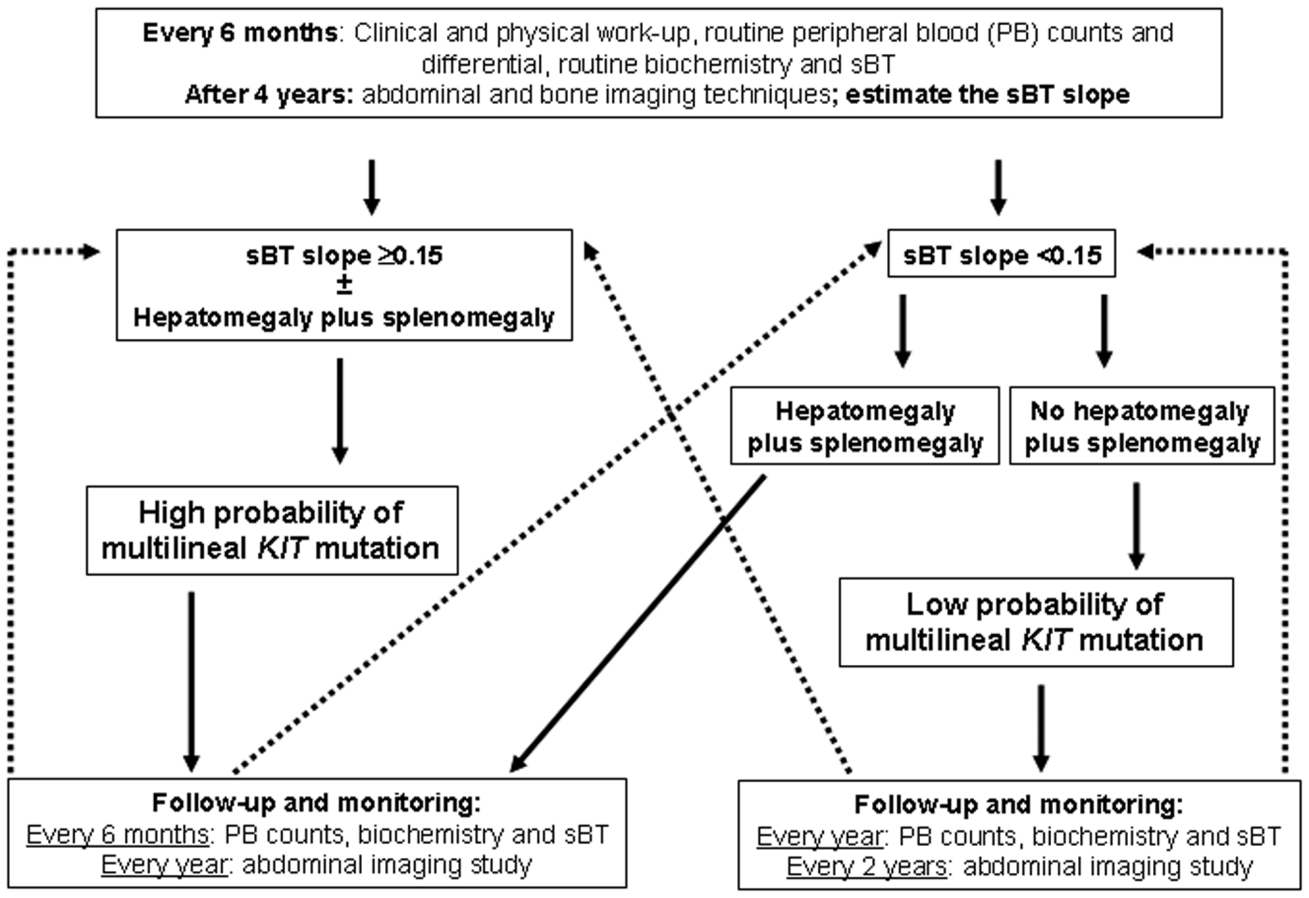
Decision-tree algorithm proposed for the follow-up of ISM patients based on sequential measurements of serum basal tryptase levels.

Altogether, these observations strengthen the value and robustness of sequential measurements of baseline serum tryptase levels, for supporting the decision-making process regarding further follow-up studies in ISM patients, and their time-points. In this regard, our proposal for centres unable to determine the pattern of *KIT* mutation in the different BM compartments of hematopoietic cells, would be to perform an initial follow-up of patients with ISM managed with conservative measures, by monitoring sBT every 6 months during the first 4 years after diagnosis, in order to estimate the sBT slope; in parallel, special attention should also be paid to the development of hepatomegaly plus splenomegaly and diffuse bone sclerosis (Figure 2). After 48 months of follow-up, every ISM patient could then be classified as having a low vs. a high probability of carrying multilineal *KIT* mutation, the latter being more prone to potentially undergo progression to more aggressive forms of the disease. ISM patients who are not suspicious of disease progression by routine criteria and who have a sBT slope <0.15 in the absence of hepatomegaly plus splenomegaly, might undergo only an yearly control with clinical and physical work-up together with a routine peripheral blood count and differential, routine biochemistry and sBT, plus an abdominal ultrasonography every 3 years (Figure 2). In contrast, ISM patients with increasing sBT levels (slope ≥0.15), should undergo peripheral blood analyses every 6 months and abdominal imaging studies every year (Figure 2). Furthermore, bone imaging techniques should be performed during follow-up for early identification of diffuse bone sclerosis, whenever, patients have bone pain or sBT values ≥100 ng/mL in the absence of other data suggesting progression to aggressive categories of the disease.

In summary, our results show that serial measurements of sBT during follow-up of ISM, is an easy accessible and powerful predictor for multilineal *KIT* mutation and disease progression, suggesting that serial sBT measurements could be of great help to assess patient prognosis, particularly in those centers where methods aimed at investigating the presence of the *KIT* mutation in different compartments of BM hematopoietic cells are not readily available We strongly recommend identificacion of ISM cases at risk of disease progression, regardless of the experience of individual centers. Only if this is done, adequate counseling, follow-up and treatment can be applied to individual patients, to avoid psychological stress, unnecessary periodical diagnostic tests and administration of cytoreductive and/or targeted therapies in the good-prognosis ISM patients.
